# An Antithrombin-Heparin Complex Increases the Anticoagulant Activity of Fibrin Clots

**DOI:** 10.1155/2008/639829

**Published:** 2008-04-14

**Authors:** Lesley J. Smith, Tracy Anne Mewhort-Buist, Leslie R. Berry, Anthony K. C. Chan

**Affiliations:** Henderson Research Centre, McMaster University, 711 Concession Street, Hamilton, L8V 1C3 Ontario, Canada

## Abstract

Clotting blood contains fibrin-bound thrombin, which is a major source of procoagulant activity leading to clot extension and further activation of coagulation. When bound to fibrin, thrombin is protected from inhibition by antithrombin (AT) + heparin but is neutralized when AT and heparin are covalently linked (ATH). Here, we report the surprising observation that, rather than yielding an inert complex, thrombin-ATH formation converts clots into anticoagulant surfaces that effectively catalyze inhibition of thrombin in the surrounding environment.

## 1. Introduction

Thrombosis results from stimulation of the coagulation pathway, which leads to thrombin cleavage of fibrinogen to form fibrin. During this process, thrombin becomes incorporated into the evolving fibrin clot and becomes a nidus for activation of coagulant factors and fibrin accretion [1]. Historically, thrombosis prevention has been through administration of heparin, which catalyzes the inhibition of thrombin by antithrombin (AT) [2]. Paradoxically, use of heparin can actually lead to protection of fibrin-bound thrombin from reaction with noncovalent AT•heparin by forming a fibrin•heparin•thrombin ternary complex [3]. Increased heparin concentrations reduce circulating prothrombotic activity but clot-associated thrombin activity reappears once heparin treatment is discontinued [3, 4]. We developed a potent nondissociable covalent complex of AT and heparin (ATH) [5] so that attraction of the heparin moiety to fibrin•thrombin would consequently neutralize thrombin via the attached AT [6]. Investigation of ATH interactions with fibrin monomer and thrombin revealed that the covalent thrombin-ATH complex formed remains adherent to fibrin through the heparin chain [6]. We were intrigued to determine if the heparin in fibrin-bound thrombin-ATH may still be able to interact with exogenous fluid-phase coagulation proteins. Thus, the present work was designed to determine if the heparin chain in the thrombin-ATH complexes bound to fibrin monomer and fibrin clot surfaces is capable of catalyzing the inhibition of free, fluid phase, thrombin by exogenously added AT.

## 2. Materials and Methods

### 2.1. Fibrin Monomer Preparation

Soluble fibrin monomer was prepared from human fibrinogen (Enzyme Research Laboratories), after removal of contaminating fibronectin by incubation of 15 mL of 130 *μ*M fibrinogen with 5 mL of gelatin agarose (Sigma) for 30 minutes. Purified fibrinogen (100 *μ*M) was incubated with human thrombin (Enzyme Research, 2 nM final) at 37°C for 4 hours, followed by centrifugation for 5 minutes at 2000 g. The fibrin polymer pellet was placed in dialysis tubing with a 12000–14000 MW cutoff and dialyzed at 4°C versus H_2_O, followed by dialysis against 0.02 M acetic acid until the fibrin dissolved. The fibrin monomer concentration was determined by absorbance at 280 nm, where a concentration of 10 mg/mL = an absorbance of 14.0.

### 2.2. Complex Preparation

Covalent antithrombin-heparin complex (ATH) was prepared by heating 1152 mg of antithrombin (AT; Affinity Biologicals Inc.) + 64 g of heparin (Sigma) in 900 mL of PBS for 14 days at 40°C. Following incubation, NaBH_3_CN was added to a final concentration of 0.05 M and the solution was further incubated at 37°C for 5 hours. Free AT was removed by making the reaction mixture 2.5 M in (NH_4_)_2_SO_4_, loading on a 1000 mL column of butyl sepharose (Amersham), washing the beads with 2.5 M (NH_4_)_2_SO_4_ and eluting the complex in 0.02 M phosphate pH 7.0. Eluent was dialyzed versus 0.01 M Tris-HCl pH 8.0 buffer and bound to 900 mL of DEAE sepharose (Amersham), followed by washing with 0.2 M NaCl in buffer and elution of purified ATH with 2.0 M NaCl in buffer. ATH was pressure-dialyzed versus PBS. The final purified ATH product possessed <5% unconjugated starting materials and contained AT which was >95% active against thrombin. Previous work has established that the AT : heparin molar ratio is essentially 1 : 1 [5].

### 2.3. Sepharose-AT Chromatography

High-affinity heparin (HAH) was prepared by loading 4 mg of heparin (in 2 mL of 0.15 M NaCl in 0.01 M phosphate pH 7.3 buffer) on a 10 mL column of sepharose-AT prepared from AT and CNBr-activated sepharose (Amersham), according to the manufacturer. The column was then washed with 0.5 M NaCl in buffer and eluted with 2 M NaCl in buffer. Recovered HAH was dialyzed against H_2_O and lyophilized.

### 2.4. Fibrin Monomer and Thrombin Inhibition

In order, 3.24 *μ*L of 37 *μ*M fibrin monomer, 2.54 *μ*L of 59 *μ*M GPRP-NH_2_ (Sigma), 1.3 *μ*L of 1 M Tris, 4.6 *μ*L 0.02 M Tris-HCl 0.15 M NaCl 0.6% polyethylene glycol 8000 pH 7.4 (TSP), 2.06 *μ*L of 1 mg/mL bovine albumin (globulin free; Sigma) in TSP, and 1.2 *μ*L of 1.08 *μ*M thrombin were mixed at 23°C in a microfuge tube. After 2 minutes, 2.06 *μ*L of either 0.125 *μ*M AT + 0.125 *μ*M heparin or 0.125 *μ*M ATH was added. Following a further 2-minute incubation, TSP solutions of excess AT (12.9 *μ*M, 1 *μ*L) and thrombin (1.08 *μ*M, 12 *μ*L) were added in rapid succession. After 2 minutes of reaction, remaining thrombin activity was determined by mixing 0.8 *μ*L of reaction mixture with 79.2 *μ*L of 0.62 mM S-2238 substrate (diaPharma, West Chester, OH, USA) in TSP, followed by neutralization after 10 minutes with 20 *μ*L of 50% acetic acid and measurement of absorbance at 405 nm. The ability to catalyze the inhibition of thrombin by AT was calculated as the percent decrease in thrombin activity relative to experiments without AT + heparin or ATH.

### 2.5. Fibrin Clots and Thrombin Inhibition

A volume of 0.5 mL of plasma was mixed with 9 *μ*L of 1 M CaCl_2_ in microfuge tubes containing 6 mm plastic loops (Fisher). After 1 hour at 37°C, clots (on loops) were removed and washed 6 times by dipping in 1 mL solutions of 0.02 M Tris-HCl 0.15 M NaCl pH 7.4 (TBS). After storage overnight at 4°C in TBS, clots were placed in TBS for 20 minutes at 23°C and 1 hour at 37°C, followed by 6 TBS washes and storage overnight at 4°C in TBS. Clots were placed in fresh TBS for 20 minutes and then incubated for 1 hour at 37°C in 1 mL of either TSP, 0.1 *μ*M AT + 0.1 *μ*M heparin; TSP, 0.1 *μ*M AT + 0.1 *μ*M HAH; or TSP, 0.1 *μ*M ATH. After washing briefly 6 times in 1 mL of TSP and once for 20 minutes in TSP at 23°C, catalytic activity of the clots was determined at 37°C in a solution of 0.5 mL 0.043 *μ*M thrombin + 0.5 mL 0.2 *μ*M AT, mixed just prior to use. Clots were dipped up and down (once per second) within the thrombin + AT solution for 1 minute. After removal of the clot, 12 *μ*L of the thrombin + AT solution was reacted with 67.2 *μ*L of 0.71 mM S-2238 in TSPfor 10 minutes at 23°C, neutralized with 20 *μ*L of 50% acetic acid and the absorbance was taken at 405 nm to determine activity. Results were calculated as a percent of thrombin activity in thrombin solutions without AT. Inhibition was determined relative to reactions without clots. Subtraction of percent thrombin activity lost in clots incubated without heparin compounds gave the net catalytic activity observed.

### 2.6. Statistical Analyses

Data were judged to be normally distributed since variance about the mean was not skewed and application of the Anderson-Darling normality test did not reveal any nonGaussian distributions. *t*-tests (2 tailed, nonpaired) were used for the fibrin monomer studies (*n* ≥6), as there were two main experimental groups. Since there were multiple groups in the fibrin clot experiments, a one-way ANOVA was performed (*n* ≥4, *P* = .004), followed by the Tukey posttest for comparisons between ATH and the other heparin experimental groups. An alpha level of 0.05 was used throughout the analyses. Data are expressed as mean ± standard error of the mean (SEM).

## 3. Results and Discussion

**Figure 1:**
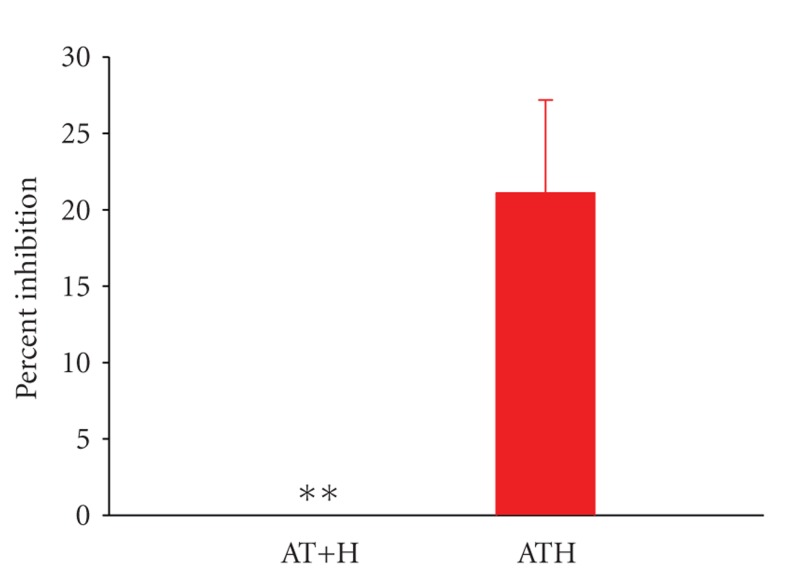
Catalysis of thrombin inhibition by anticoagulant bound to purified fibrin. Thrombin on fibrin was mixed with antithrombin (AT) + heparin (H) or covalent antithrombin-heparin (ATH) and ability to accelerate reaction of additional thrombin + AT determined as the decrease in percent thrombin activity remaining compared to experiments without H or ATH. Data are expressed as mean ± SEM and *n* ≥ 6. **P < .01, using a *t*-test relative to ATH.

Initial experiments were conducted to ensure that thrombin on fibrin was neutralized by ATH and that the inhibited surface did not protect additional free thrombin from attack by fluid-phase AT. Results from chromogenic assays showed that fibrin•thrombin-ATH had no effect on soluble thrombin functional activity. While all thrombin on fibrin was capable of reaction with ATH, a rapid inactivation of supplemental thrombin ensued in the presence of exogenous AT. Careful studies confirmed that, compared to fibrin alone, thrombin-ATH on fibrin significantly catalyzed inhibition of excess thrombin by AT (*P* = .007) while similar tests with noncovalent AT + heparin mixtures demonstrated no effect (Figure 1).

**Figure 2:**
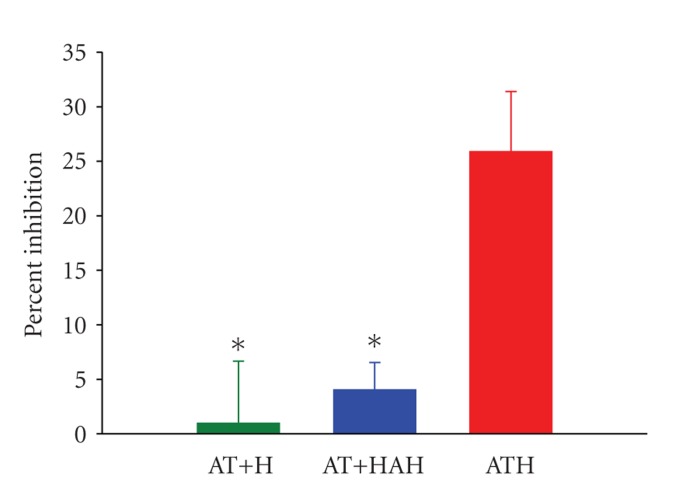
Catalysis of thrombin inhibition by anticoagulant bound to fibrin clots. Recalcified plasma clots were incubated with antithrombin (AT) + heparin (H), AT + high AT-affinity heparin (HAH) or antithrombin-heparin covalent complex (ATH), and increase in percent inhibition of soluble thrombin by AT calculated relative to clots in buffer. Data are expressed as mean ± SEM and *n* ≥ 4. *P < .05, using the Tukey test (after one-way ANOVA) for determination of significant differences relative to ATH.

We next examined the capacity for surface anticoagulation of ATH in a physiological system. Clots from recalcified plasma were treated with buffer or small amounts of ATH. Incubation of thrombin + AT solutions with ATH-neutralized clots showed strong inhibition of enzyme activity relative to AT + heparin or AT + heparin with high affinity for AT (HAH), with *P* values of 0.026 and 0.023, respectively (Figure 2). Moreover, insignificant effects on soluble thrombin + AT were observed for clots treated with either AT + heparin or AT + HAH (95% confidence intervals of −17.2–19.1 and −2.2–10.2, resp.). Thus, we have demonstrated that not only does ATH inactivate clot prothrombotic activity, but the surface produced on the clot also contains heparin which maintains anticoagulant activity in the surrounding milieu. This finding is in contrast to that with heparin, where heparin absorbed on procoagulant clots was ineffective at influencing thrombin/AT reactions (Figure 2). Furthermore, the lack of enhancement by HAH of thrombin reaction with AT (Figure 2) confirmed that enrichment of high-affinity AT binding sites in the heparin chains of clot-bound ATH [5] was not responsible for ATH's superior catalytic properties.

**Figure 3:**
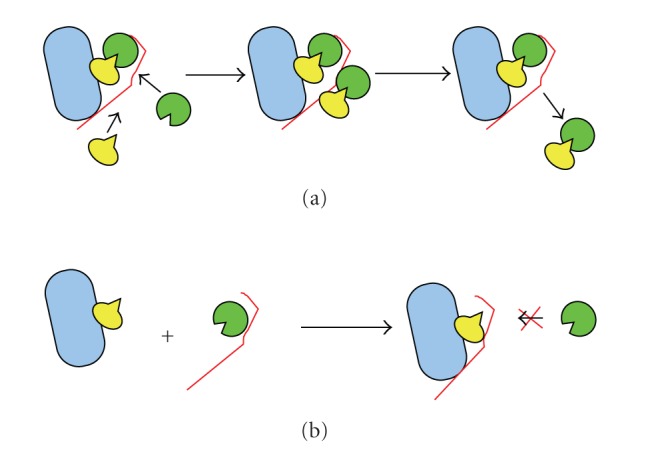
Model of catalysis of thrombin inhibition by fibrin-bound antithrombin-heparin covalent complex (ATH). The fibrin (blue) clot-bound thrombin-ATH (thrombin in yellow, antithrombin (AT) in green, heparin in red) is able to catalyze thrombin inhibition by AT since the heparin chain is available to bind both free thrombin and AT (panel (a)). In the case of noncovalently linked heparin, the heparin-catalyzed inhibition of thrombin by AT is negated through the formation of a fibrin-thrombin-heparin ternary complex (panel (b)).

A model (Figure 3) shows the thrombin-ATH complex, noncovalently tethered through heparin to fibrin, which is catalyzing reaction of incoming thrombin + AT via conventional heparin template bridging [7]. This mechanism is consistent with data showing that ATH causes reduction in clot size within injured vessels, compared to clot growth which occurs with AT + heparin [8]. Our findings have implications that could alter treatment paradigms. By “coating” fibrin-thrombi with ATH, clinicians may use clots as platforms to pacify hyperthrombotic activity in damaged vasculature.

## 4. Conclusions

Covalent linkage of AT to heparin facilitates formation of fibrin clot-bound structures with heparin moieties possessing potent thrombin-inhibitory catalytic activity. Conversion of clot surfaces into functional anticoagulants may be done selectively since ATH heparin chains are electrostatically attracted to fibrin•thrombin [6]. In vivo experiments will confirm the utility of anticoagulated thrombi in treatment models.

## Abbreviations

AT: antithrombin,
ATH: covalent antithrombin-heparin complex,
H: heparin,
HAH: heparin with high affinity for antithrombin,
TSP: tris-saline-polyethylene glycol buffer,
TBS: tris-buffered saline,
SEM: standard error of the mean.
